# Mining Temporal Protein Complex Based on the Dynamic PIN Weighted with Connected Affinity and Gene Co-Expression

**DOI:** 10.1371/journal.pone.0153967

**Published:** 2016-04-21

**Authors:** Xianjun Shen, Li Yi, Xingpeng Jiang, Tingting He, Xiaohua Hu, Jincai Yang

**Affiliations:** 1 School of Computer, Central China Normal University, Wuhan, China; 2 College of Computing and Informatics, Drexel University, Philadelphia, United States of America; Peking University Health Science Center, CHINA

## Abstract

The identification of temporal protein complexes would make great contribution to our knowledge of the dynamic organization characteristics in protein interaction networks (PINs). Recent studies have focused on integrating gene expression data into static PIN to construct dynamic PIN which reveals the dynamic evolutionary procedure of protein interactions, but they fail in practice for recognizing the active time points of proteins with low or high expression levels. We construct a Time-Evolving PIN (TEPIN) with a novel method called *Deviation Degree*, which is designed to identify the active time points of proteins based on the deviation degree of their own expression values. Owing to the differences between protein interactions, moreover, we weight TEPIN with connected affinity and gene co-expression to quantify the degree of these interactions. To validate the efficiencies of our methods, ClusterONE, CAMSE and MCL algorithms are applied on the TEPIN, DPIN (a dynamic PIN constructed with state-of-the-art three-sigma method) and SPIN (the original static PIN) to detect temporal protein complexes. Each algorithm on our TEPIN outperforms that on other networks in terms of match degree, sensitivity, specificity, F-measure and function enrichment etc. In conclusion, our *Deviation Degree* method successfully eliminates the disadvantages which exist in the previous state-of-the-art dynamic PIN construction methods. Moreover, the biological nature of protein interactions can be well described in our weighted network. Weighted TEPIN is a useful approach for detecting temporal protein complexes and revealing the dynamic protein assembly process for cellular organization.

## Introduction

Cellular processes are typically carried out by protein complexes formed by groups of proteins interacting with each other, rather than by individual protein. Large-scale protein-protein interaction data being produced along with high-throughput techniques such as yeast two-hybrid (Y2H) provide maps of molecular networks for several organisms [1], thereby promoting the emergency of many computational algorithms for identifying protein complexes from protein-protein interaction network (PIN). Most of these methods are based on solely network clustering [2–5] or integrated with multiple biological data [6–9]. Identifying protein complex has significant implications in revealing the important principle of protein organization within cell [10, 11].

While significant progress has been made in those computational analysis of proteome-scale cellular networks, the inherent dynamics of protein interactions within these networks are often overlooked [12]. Cellular systems are highly dynamic and responsive to the stimulus from external environment—the biomolecules and their interactions are changing over time, environment and different stages of cell cycle [12]. Temporal protein complexes are typically formed by the dynamic assembly and disassembly of proteins to implement various biological functions. Systematically analyzing the temporal protein complexes can not only improve the accuracy of protein complexes identification but also strengthen our biological understanding on the dynamic protein assembly process for cellular organization [13]. Undoubtedly, the shift from static interactome to dynamic protein complexes plays an important role in uncovering the dynamic organization characteristics in cell systems [14]. The dynamic evolutionary procedure of protein interactions in the real world can be reflected in dynamic PIN, thus it provides a reliable foundation for mining temporal protein complexes with more effectiveness. Besides, dynamic PIN conduces to illustrate how the onset and progression of disease are reflected in the time-evolving protein interaction network, and contributes to the detection of a disease prior to the development of clinical symptoms, thus paving a way to preventative treatment [15].

Nevertheless, the protein interaction networks derived from high throughput processing techniques could not enable us to discern temporal and contextual signals. Fortunately, gene expression data provide a complementary view by their ability to monitor changes in RNA concentration in thousands of genes simultaneously [16, 17]. Thus we can construct time-evolving dynamic PIN with these data to detect temporal protein complexes. Yet, how to recognize the activities of proteins is the key issue to construct dynamic PIN.

De Lichtenberg et al. constructed a dynamic PIN over the yeast mitotic cell cycle [18]. For the periodically expressed proteins, they appear at the time point of peak expression; while for the non-periodically expressed proteins, they present at every time point. As a result, only 300 proteins are involved in this dynamic PIN in contrast to nearly 5000 proteins in yeast proteome. Tang et al. adopted a recommended threshold to filter expression noises from the gene expression profiles over three successive metabolic cycles [16]. Further, they constructed a time-series protein interaction network (called TC-PIN), which cover 14904 interactions among 3520 proteins (about 70%) in average. TC-PIN has better performance than the original static PIN in practice of predicting protein complexes; however, without considering the differential expression levels of different genes, the proteins with low expression peak filtered by a relatively higher threshold will be improperly missed in TC-PINs, which will cause the inaccurate analysis of dynamic PIN [17]. Rather than employing a global threshold to determine a protein’s activity, Wang et al. designed a three-sigma method to identify the active time points of each protein by considering its own characteristic expression curve [17]. Based on two different gene expression profile sets, namely GSE3431 and GSE4987, the authors constructed two dynamic protein interaction networks (called DPINs) with smaller scales, on which the protein complex predictions have been proved to be better than those on TC-PINs and static PIN [17]. Observation on these time-evolving dynamic PINs suggests that the network scale and density can be used to measure the quality of different dynamic networks derived from the same data sets [15]. Though three-sigma method has been a state-of-the-art approach for constructing dynamic PIN and has been widely accepted in academic circle [13], it has its shortcomings—many proteins with high expression levels would be involuntarily filtered out by their high active thresholds. This would cause the unconvincing analysis of dynamic PIN.

We propose a novel method—*Deviation Degree*, to recognize the active time point of protein according to the deviation degree of its expression values from their arithmetic average. Then a time-evolving protein interaction network (called TEPIN) is constructed by mapping active proteins into the original static PIN. TEPIN could more closely imitate the dynamic evolutionary procedure of protein interaction. Experimental results show that TEPIN greatly improves the prediction of temporal protein complexes in terms of match degree, sensitivity, specificity, F-measure and function enrichment, which indicates that our method not only successfully surmounts the drawback mentioned above, but also outperforms three-sigma method in practice for recognizing protein activity.

Further, we present a weighted approach for PINs, which is applied on TEPIN to quantify the degree of interactions among proteins based on connected affinity [19] and gene co-expression [8]. It has been noticed that the interactions among proteins should not be treated equally, but the difference among these interactions cannot be reflected at all in PINs. The protein interaction data produced by high-throughput experiments are not absolutely convincing. There exist a huge amount of false positive interactions and some transient interactions cannot be captured due to the limitations of current experimental techniques. Our weighted strategy can not only describe the biological nature of protein interactions, but also provide an approach for reducing the impacts of the inherent false positives and false negatives within PINs. Experimental results indicate that the weighted TEPIN further optimizes the identification of temporal protein complexes with aspect of various evaluation metrics.

## Materials and Methods

### Experimental Data

Protein interaction network: We use yeast protein interaction data derived from DIP [20] (Version of 20101010), which contains all the interactions of proteins from a particular species and provides species-specific subsets. The static PIN includes 24743 interactions among 5093 distinct proteins after removing the self-interactions and repeated ones.

Gene expression data: Gene expression data over three successive metabolic cycles are available from GEO (Gene Expression Omnibus) [21] with accession number GSE3431. This dataset includes the expression profiles of 9335 probes under 36 different time points. The gene products involved in the gene expression data cover 97.8% of the proteins in the static PIN.

Known protein complex dataset: MIPS Complex-Catalogue is probably one of the most comprehensive public datasets of yeast complexes available and allows precise standardized functional descriptions of genes [22]. It is often used as the benchmarks to evaluate protein complex prediction [5, 19]. We thus derive the known yeast protein complexes from MIPS (ftp://ftpmips.gsf.de/), which contains 1063 protein complexes through a series of preprocessing, excluding the ones containing only one protein.

### Active Time Points of Proteins and TEPIN

Typically, the dynamics of protein interactions are indirectly reflected in the active time points of proteins. Therefore, the construction of a time-evolving dynamic PIN is determined by the identification of these active time points.

#### Identification of the Active Time Points of Proteins

The expression values of each gene/protein fluctuate in a certain range, meaning they rise and fall around their arithmetic average value. *Deviation Degree* is a method created to identify the active time points of each protein according to the deviation degree of its expression values from their arithmetic average. For a gene *i* at time point *t* (*t*∈{1,2,…,*n*}), only if the positive deviation degree of its expression value at this time point is greater than the standard deviation of the gene’s expression values over time points 1 to *n*, we consider it to be active at this time point. Let *exp*_*it*_ denote the expression value of gene *i* at time point *t*, then the gene’s arithmetic average (*u*_*i*_) and standard deviation (*σ*_*i*_) of its expression values over time points 1 to *n* can be formulated as Eqs (1) and (2). Therefore, we define the active threshold for protein/gene *i* as Eq (3). A protein is considered to be active at the time points with expression values that are above its active threshold value.

$$u_{i}=\frac{1}{n}\sum_{t=1}^{n}{\text{exp}}_{it}$$ (1)

$$\sigma_{i}^{2}=\frac{1}{n-1}\sum_{t=1}^{n}{\left({\text{exp}}_{it}-u_{i}\right)}^{2}$$ (2)

$$ActiveThreshold(gene_{i})=u_{i}+\sigma_{i}$$ (3)

Where *n* is 36. We manage to achieve the time-evolving active protein sets under *n* time points. To begin with, each protein’s active threshold value is calculated according to (3). Then, for a time point *t* (*t*∈{1,2,…,*n*}), each of the proteins is determined to be active or not by comparing its expression value at this time point with its active threshold value. As a result, we obtain the active protein set at time point *t*, which is denoted as *ActiveProteins*_*Tt*_. After the traversal of *n* time points, a sequential collection containing *n* active protein sets is generated, which is denoted as {*ActiveProteins*_*T1*_,…, *ActiveProteins*_*Tn*_}.

When a single global threshold is used to identify proteins’ active time points, the proteins with low expression levels will be filtered out even if they are always active during the whole metabolic cycle; while the ones with high expression levels during the whole metabolic cycle will be considered as active proteins at all the time points, even if their activities never appear. Although three-sigma method overcomes these drawbacks [17], it brings another problem—the proteins with high expression levels should be filtered out by their high active threshold values. However, these disadvantages are eliminated in our *Deviation Degree* method, which is capable to recognize the active time points of proteins correctly, including the ones with very low or high expression levels.

#### Construction of TEPIN

Actually, the PINs in the real world are changing over time, environment and different stages of cell cycle. The assembly processes of almost all eukaryotic complexes are just-in-time, contrary to the just-in-time synthesis observed in bacteria [23]. Just-in-time assembly means that most subunits of a complex are pre-transcribed, while some units are transcribed when required to assemble the final complex [23].

As the gene products involved in the gene expression data cover 97.8% of the proteins in the original static PIN, it is reasonable to construct TEPIN by combining these two datasets. A TEPIN behaves as *n* snapshots, each of which is a subset of the original static PIN. For a time point *t* (*t*∈{1,2,…,*n*}), the proteins in *ActiveProteins*_*Tt*_ and their interactions in static PIN are reserved to form a temporal *PIN*_*Tt*_, namely, a snapshot of the dynamic PIN at time point *t*. After the traversal of *n* time points, we generate a TEPIN which is denoted as a sequential collection {*PIN*_*T1*_,…,*PIN*_*Tn*_}. TEPIN reveals the dynamic evolutionary procedure of protein interactions.

### Weighted Approach Based on Connected Affinity and Gene Co-expression

In this section, TEPIN is converted into a weighted network in which the edge-weights represent the degree of protein interactions.

For the one hand, it has been noticed that the interactions among proteins shouldn’t be treated equally. But owing to the neglect of biological nature, only Boolean values “1” and “0” can be employed to denote whether two proteins could interact or not in PIN. To resolve this issue, Li et al. defined connected affinity coefficient (*CAC*) to enhance the biological character of PIN [19]. According to Li et al., for a protein complex including proteins *P*_*i*_ and *P*_*j*_, their relationship *RP*_*ij*_ should be closer when the complex contains more proteins but slighter when it includes more interactions [19]. Thus Connected Coefficient *CC*_*ij*_ standing for how large possibility to connect the proteins *P*_*i*_ and *P*_*j*_ in one protein complex is defined for *RP*_*ij*_ as Eq (4):

$$CC_{ij}=\frac{N_{k}}{R_{k}}$$ (4)

Where *N*_*k*_ and *R*_*k*_ represent the number of proteins and interactions within protein complex *k* respectively. Considering the fact that proteins interacting with each other are often subordinate more than one complex simultaneously, Connected Affinity Coefficient *CAC*_*ij*_ standing for the likelihood of that two proteins *P*_*i*_ and *P*_*j*_ could interact with each other is inferred from a protein complex set [8]:

$$CAC_{ij}=\sum_{k=1}^{M_{ij}}CC_{ij}$$ (5)

Where *M*_*ij*_ is the number of the known protein complexes which include the interaction connecting *P*_*i*_ and *P*_*j*_. The value of *CAC*_*ij*_ thus depends on two factors: the number of the protein complexes including interaction *RP*_*ij*_ and their individual values of *CC*_*ij*_.

For the purpose of validating the effectiveness of CAC, Li et al. split the known protein complexes into training set and testing set in their previous work, and the comparison between identified complexes and benchmarks in testing set has already demonstrated that the incorporation of CAC provides powerful support to reveal the biological properties in PINs [19]. For this reason, in our experiments there is no need to make a duplication of effort on splitting the benchmarks into two catalogs, namely training set and testing set. Therefore, we calculate CAC with all the known protein complexes as a part of the weight in PIN to generate more helpful biological knowledge.

For the other hand, the protein interaction data are not absolutely convincing due to the limitations of the associated experimental techniques. Interestingly, the integration of gene co-expression—which is usually measured by Pearson Correlation Coefficient (*PCC*)—can diminish the impacts of the inherent false negatives and false positives in PINs [8]. For two columns of gene expression profiles *x* = (*x*_1_,…, *x*_*n*_) and *y* = (*y*_1_,…, *y*_*n*_). *PCC*_*xy*_ can be denoted as Eq (6):

$$PCC_{xy}=\frac{\sum_{i=1}^{n}\left(x_{i}-\bar{x})(y_{i}-\bar{y}\right)}{\sqrt{\sum_{i=1}^{n}{\left(x_{i}-\bar{x}\right)}^{2}}\sqrt{\sum_{i=1}^{n}{\left(y_{i}-\bar{y}\right)}^{2}}}$$ (6)

Where $\bar{x}$ and $\bar{\text{y}}$ represent the average expression values of gene *x* and gene *y* respectively. The values of *PCC*_*xy*_ range from -1 to 1.

To characterize effectively the biological nature of protein interactions, we weight TEPIN by combining *CAC* with *PCC*. For each pair of proteins *P*_*i*_ and *P*_*j*_ that interact with each other, we take the sum of *CAC*_*ij*_ and *PCC*_*ij*_ as the weight of interaction *RP*_*ij*_:

$$W_{ij}=CAC_{ij}+PCC_{ij}$$ (7)

*CAC*_*ij*_ and *PCC*_*ij*_ are complementary and consistent with each other. First, Due to the incompleteness of known protein complex data and the false negatives of protein interaction data, some of the interactions will gain lower weight. In this case, it is reasonable to increase the weight with positive *PCC*_*xy*_ which means gene *x* and gene *y* are co-expressed; Instead, some interactions will gain higher weight because of the false positives of interactions and the fact that the known protein complex set contains some putative ones determined by high-throughput experiments. So it is also reasonable to decrease the weight with negative *PCC*_*xy*_ which denotes the two genes’ expressions are inhibited with each other. Second, the higher degree of the interaction between two proteins, the greater the likelihood that they participate in the same biological functions, thus the greater the values of both *CAC*_*ij*_ and *PCC*_*ij*_.

Our weighted approach is applied on each temporal *PIN*_*Tt*_ (*t*∈{1,2,…,*n*}) of TEPIN, thereby generating a weighted TEPIN denoted as {*WDPIN*_*T1*_,…,*WDPIN*_*Tn*_}. Interactions with positive weight are deemed to be positive interactions and reserved within weighted PIN, while the others are eliminated as false positives. The ratio of eliminated interactions varies between 0.021 to 0.134 (mean = 0.090, standard deviation = 0.039) across 36 time points.

### Mining Temporal Protein Complexes

As we shall demonstrate in that following section, WTEPIN provides a more reliable basis for detecting temporal protein complexes. As three-sigma method has been demonstrated to be superior to the other dynamic PIN construction methods and has been widely accepted in academic circle as a state-of-the-art method to date [13], it is used to evaluate the validity of our *Deviation Degree* method. To accomplish this goal, we employ several classic and state-of-the-art algorithms to mine protein complexes from our TEPIN, DPIN (constructed with three-sigma method based on the same datasets) and SPIN. Markov Cluster algorithm (MCL) [3], which is more tolerant to noise and behaves more robustly than other classic algorithms, has been widely used to analyze complex networks. ClusterONE [4] and CAMSE (connected affinity and multi-level seed extension) [5] are two state-of-the-art algorithms designed for identifying protein complexes. Cytoscape [24] is a famous open source software platform on which we can conveniently perform ClusterONE algorithm on protein interaction networks, thus we employ it to produce protein complexes. Considering the high efficiencies of these three algorithms, we employ them to compare the performances of various kinds of networks involved in this study. Given a dynamic PIN, an algorithm performs separately on *n* temporal snapshots. Therefore, *n* groups of predicted protein complex are generated, which are finally merged into one group. The predicted protein complexes containing only one protein will be wiped out. Besides, inner kernel extension threshold and outer kernel extension threshold involved in CAMSE algorithm need to be adjusted to render the best performance.

We need to filter the redundant complexes from predicted protein complex set due to the high overlap ratio within them. To be more specific:

1. All the predicted protein complexes are sorted in descending order by their size;
2. For each of the undiscarded protein complexes *C*_*u*_, we compare it separately to the other undiscarded ones with smaller or the same size (denoted by {*C*_*o*_}). Among the complexes in {*C*_*o*_}, the one whose similarity with *C*_*u*_ is greater than a very high similarity threshold will be discarded.

Such a filter operation reduces the number of predicted protein complexes and retains the correct ones, which is helpful to the analysis of experimental results. The similarity threshold is set to 1.0 for ClusterONE and MCL algorithms, and 0.8 for CAMSE algorithm [5, 16].

### Metrics for Evaluating Identified Protein Complexes

Overlapping Score (*OS*) [9] Eq (8) is often used to assess the match degree between a predicted protein complex *pc* and a known protein complex *kc*:

$$OS(pc,kc)=\frac{{\left|pc\cap kc\right|}^{2}}{\left|pc\right|\times \left|kc\right|}$$ (8)

Where |*pc*∩*kc*| represents the number of the proteins involved in both complexes *pc* and *kc*; |*pc|* and *|kc|* represent the number of proteins involved in complex *pc* and complex kc respectively. Two protein complexes are considered to be matched if their overlapping score is greater than or equal to a given threshold, which is set to 0.2, the same as many other researches [9]. Particularly, *OS*(*pc*,*kc*) = 1 indicates that the two complexes *pc* and *kc* match perfectly. The predicted protein complex sets identified from various networks are separately compared against the known protein complex set.

Sensitivity (*Sn*) and Specificity (*Sp*) are typically employed to evaluate the detection of protein complexes [19]. Let true positives (*TP*) denote the number of predicted protein complexes that match with known complexes, false positives (*FP*) denote the number of unmatched predicted complexes, and false negatives (*FN*) denote the number of known protein complexes which match with none of the predicted protein complexes, then *Sn* and *Sp* can be defined as Eqs (9) and (10), respectively. The harmonic mean of *Sn* and *Sp*, also known as *F-measure* Eq (11), is often used to assess the overall accuracies of various methods [9].

Sn=TP/(TP+FN) (9)

Sp=TP/(TP+FP) (10)

$$F-measure=\frac{2\times Sn\times Sp}{Sn+Sp}$$ (11)

Larger *Sn* to some extent indicates that more known protein complexes could be recognized, while higher *Sp* shows that higher percentage of predicted protein complexes match with known protein complexes.

To evaluate the statistical significance of the identified protein complexes, many researchers annotate their main biological functions by using *p-value* formulated as Eq (12) [16, 17]. Given a predicted protein complex containing *C* proteins, *p-value* calculates the probability of observing *k* or more proteins from the complex by chance in a biological function shared by *F* proteins from a total genome size of *N* proteins [25]:

$$p-value=1-\sum_{i=0}^{k-1}\frac{\left(\begin{array}{c} F\\ i \end{array}\right)\left(\begin{array}{c} N-F\\ C-i \end{array}\right)}{\left(\begin{array}{c} N\\ C \end{array}\right)}$$ (12)

The lower the *p-value* is, the stronger biological significance the complex possesses, while the complex with *p-value* greater than 0.01 is deemed to be meaningless at all. Generally speaking, the larger protein complexes possess the smaller *p-values*.

## Results and Discussion

### Analysis of Network Properties

First of all, we analyze the properties of three kinds of networks—TEPIN, DPIN and SPIN (static PIN)—in terms of the average scale and network density. As is shown in Table 1, in contrast to SPIN, the sizes of TEPIN and DPIN are greatly decreased while their network densities are markedly increased, which is mainly due to the fact that dynamic PINs eliminate the noises which exist in static PIN. Moreover, the average scale of TEPIN is evidently smaller than that of DPIN, while the average density of TEPIN is approximately two times to that of DPIN. Therefore, the probability that the proteins interacting with each other in our TEPIN share the same or similar biological functions is greater than that in DPIN and SPIN.

**Table 1:** The properties of TEPIN, DPIN and SPIN.

Network	Average nodes	Average edges	Average density
**TEPIN**	447	839	0.008800675
**DPIN**	609	917	0.004794229
**SPIN**	5093	24743	0.001908184

**Fig 1:**
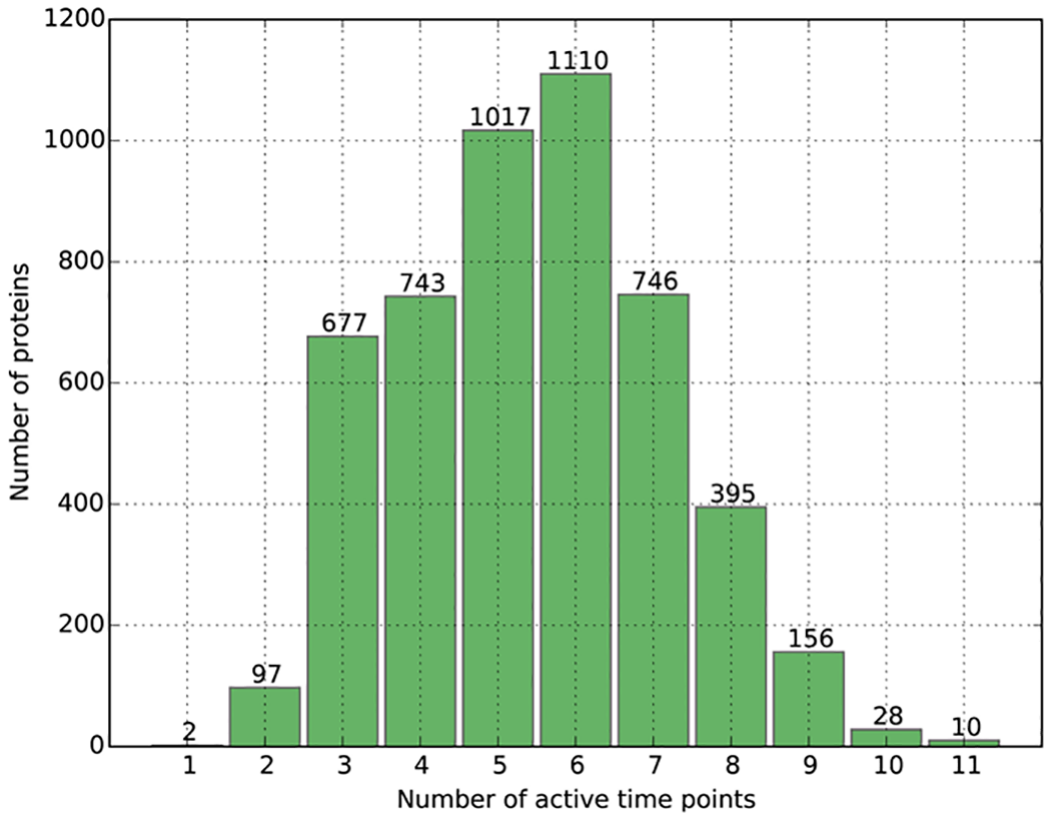
Distribution of the number of proteins with varying amount of active time points in TEPIN.

Fig 1 exhibits the distribution of the number of proteins with varying amount of active time points in TEPIN. For example, 1110 proteins are active at 6 time points, while only 2 proteins are active at just one time point. It can be seen that the numbers of active time points of most proteins (94.1%) range from 3 to 8, explaining they are active in the time of one forth to two thirds of a metabolic cycle.

In the rest of this section, we’ll confirm the validity of our TEPIN and its weighted strategy by assessing their overall performances with three classic evaluation metrics (See Materials and Methods).

### Comparison with the Known Protein Complexes

#### Validity of TEPIN

**Fig 2:**
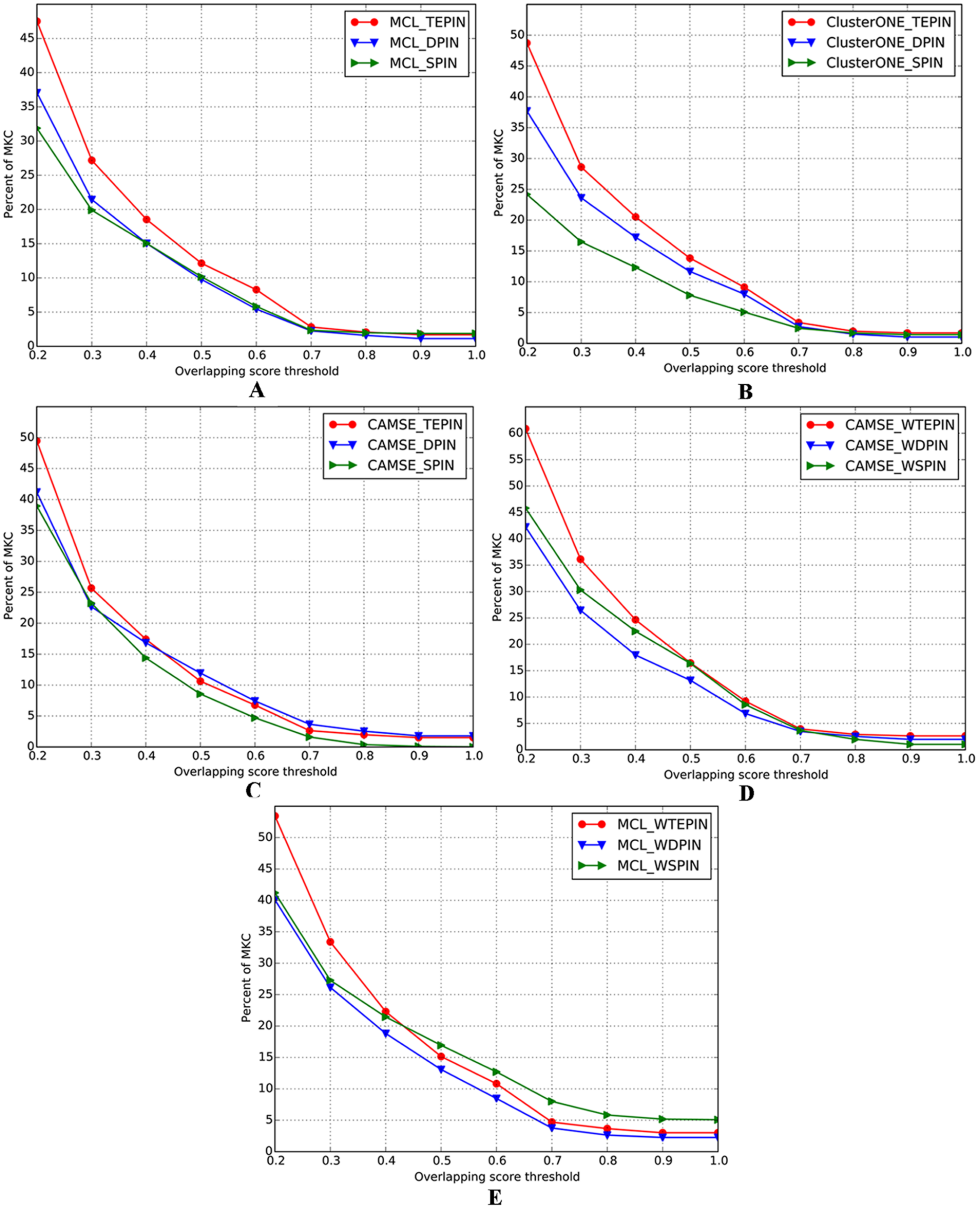
Percentage comparison of known protein complexes matched by the predicted protein complexes detected from various kinds of networks.

To validate the effectiveness of our constructed TEPIN, we implement the percentage comparison of the matched known protein complexes when applying MCL, CAMSE and ClusterONE algorithms to SPIN, DPIN and TEPIN. As is shown in Fig 2, the fraction of matched known protein complexes on TEPIN are evidently higher than that on SPIN and DPIN when *OS* threshold ranges from 0.2 to 0.4. Particularly, MCL algorithm obtains 47.5% as its percentage from TEPIN, which is 28% and 49% greater than that achieved from DPIN and SPIN respectively as *OS* threshold is set to 0.2 (see Fig 2(A)); ClusterONE algorithm obtains 48.7% as its percentage from TEPIN, which advances 29% and 102% in contrast to DPIN and SPIN respectively (see Fig 2(B)); CAMSE algorithm achieves 49.5% as its percentage from TEPIN, which is 20% and 27% higher than that obtained from DPIN and SPIN respectively (see Fig 2(C));.

In addition, the comparisons between weighted networks further illustrate the advantage of WTEPIN when we perform MCL and CAMSE algorithms (ClusterONE algorithm does not apply to weighted networks in cytoscape platform), which is shown in (Fig 2D and 2E). The fractions of matched known protein complexes on WTEPIN are evidently higher than those on WSPIN and WDPIN when *OS* threshold ranges from 0.2 to 0.4. Particularly, CAMSE obtains 60.9% as its percentage from WTEPIN, which advances 44% and 33% in contrast to WDPIN and WSPIN respectively (see Fig 2(D)) at *OS* threshold 0.2; while MCL obtains 53.4% as its percentage from WTEPIN, which advances 33% and 30% in contrast to WDPIN and WSPIN respectively (see Fig 2(E)). TEPIN is capable to describe the dynamics of protein interactions more effectively than DPIN, which contributes to the improvements of protein complex detection.

**Fig 3:**
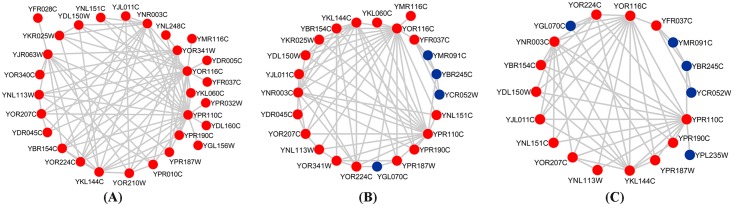
The protein complexes labeled as 550.1.213 predicted from WTEPIN and WDPIN. (A) shows the real complex labeled as 550.1.213 in the known protein complex set. (B) and (C) are the protein complexes with the identical label predicted from WTEPIN and WDPIN by CAMSE algorithm respectively. For each predicted protein complex, the proteins shown in red are involved in the real complex, while those shown in blue are not.

More interestingly, Fig 3 illustrates an example of a protein complex labeled as 550.1.213, which is more similar to the protein complex with the identical label identified from WTEPIN, rather than the one identified from WDPIN. In this illustration, the real complex consists of 29 proteins, of which 19 proteins are covered in the complex labeled as 550.1.213 identified from WTEPIN (see Fig 3(B)), while only 14 proteins are covered in the one that identified from WDPIN (see Fig 3(C). The overlapping score between the real protein complex and these two predicted protein complexes are 0.541 and 0.355 respectively, which explains the prediction on our WTEPIN is more accurate than that on WDPIN. Meanwhile, observation on the proteins uninvolved in the real protein complex (shown in blue) shows that there is one more protein within the complex identified from WDPIN: *ypl235w* to which only one protein node connects. In addition, these three protein complexes share the identical Gene Ontology terms such as RNA polymerase activity | AmiGO with *p-values* 5.01e-45 (Fig 3(A)), 3.16e-37 (Fig 3(B)) and 7.13e-32 (Fig 3(C)) respectively. Therefore, this example suggests that our WTEPIN can reflect the dynamics of protein interaction network more realistic, which makes the prediction of protein complexes more correctly.

#### Validity of weighted approach

**Fig 4:**
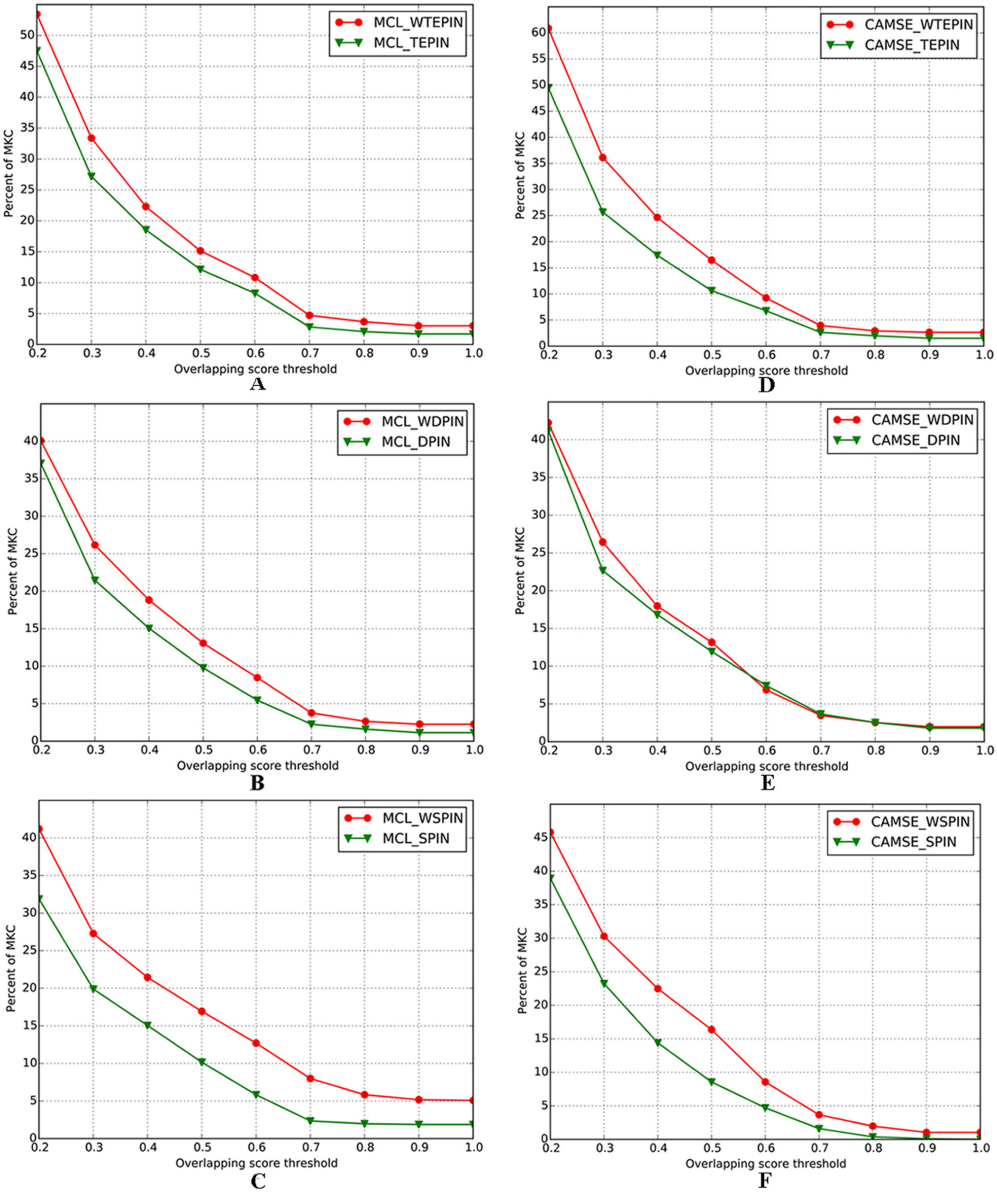
Percentage comparison of known protein complexes matched by the predicted protein complexes detected from unweighted and weighted networks.

Fig 4 exhibits the performance comparison of weighted and unweighted networks under varying *OS* threshold. It can be seen that the weighted networks evidently outperform the corresponding unweighted ones. For instance, when we set *OS* threshold to 0.3, the percentages obtained from WTEPIN, WDPIN and WSPIN by MCL algorithm are 22%, 23% and 37% higher than that achieved from TEPIN, DPIN and SPIN, respectively (see (Fig 4A, 4B and 4C)); the fractions achieved from WTEPIN, WDPIN and WSPIN by CAMSE algorithm are 41%, 17% and 30% higher than that obtained from TEPIN, DPIN and SPIN, respectively (see (Fig 4D, 4E and 4F)). In short, owing to the fact that the biological properties of the protein interactions are well reflected in the weighted networks, the predictions of protein complexes get significantly optimized.

### Measurements of Sensitivity and Specificity

#### Performance of TEPIN

We use several metrics for evaluating the performance of TEPIN including Sensitivity, Specificity and F-measure (See Materials and Methods). Table 2 shows the overall performance comparison of SPIN, DPIN and our TEPIN. Applying CAMSE algorithm, we predict 2906 protein complexes with an average size of 9 proteins from WTEPIN, of which 1599 match with known protein complexes; 647 known protein complexes are successfully detected from WTEPIN, while only 487 ones can be identified from WSPIN. Moreover, the numbers of protein complexes detected from (W)TEPIN are almost greater than those detected from (W)DPIN or (W)SPIN, meaning our new method can detect more new knowledge. As is shown in Table 2, our TEPIN always outperforms DPIN and SPIN. For instance, CAMSE obtains the highest *Sn* 0.794 and *F-measure* 0.650 from WTEPIN; MCL achieves 0.481 as its *F-measure* from WTEPIN, which is 6% and 46% higher than that achieved from WDPIN and WSPIN respectively; in addition, MCL achieves 0.353 as its *F-measure* from TEPIN, which is 12% and 49% higher than that achieved from DPIN and SPIN respectively; ClusterONE algorithm also achieves the highest *Sn* and *F-measure* from TEPIN. Although the values of *Sp* obtained from TEPIN (WTEPIN) are little lower, which is mainly due to their higher #*PC*, the values of *MKC* are always greater than those achieved from DPIN (WDPIN). Obviously, our *Deviation Degree* method is superior to the state-of-the-art three-sigma method in practice for recognizing the active time points of proteins.

**Table 2:** Performance comparison of SPIN, DPIN and TEPIN.

Algorithms	Networks	#AS	#PC	#MPC	MKC	*Sn*	*Sp*	*F-measure*	#Perfect
**CAMSE**	WTEPIN	9.0	2906	1599	647	0.794	0.550	0.650	28
	WDPIN	9.8	2893	1389	449	0.693	0.480	0.567	21
	WSPIN	8.7	1150	734	487	0.560	0.638	0.597	11
	TEPIN	4.9	2401	925	526	0.633	0.385	0.479	16
	DPIN	4.3	2433	967	468	0.607	0.397	0.480	19
	SPIN	8.7	1274	586	414	0.474	0.460	0.467	0
**MCL**	WTEPIN	5.6	1630	672	568	0.576	0.412	0.481	32
	WDPIN	5.7	1317	576	426	0.475	0.437	0.455	24
	WSPIN	3.9	741	269	438	0.301	0.363	0.329	54
	TEPIN	5.8	2276	608	505	0.521	0.267	0.353	18
	DPIN	6.9	2202	538	394	0.446	0.244	0.316	12
	SPIN	5.1	957	226	340	0.238	0.236	0.237	20
**ClusterONE**	TEPIN	3.5	1744	583	518	0.517	0.334	0.406	18
	DPIN	3.8	1689	598	400	0.474	0.354	0.405	11
	SPIN	3.7	613	180	257	0.183	0.294	0.225	15

#AS: the average size of predicted protein complexes;

#PC: the total number of predicted protein complexes;

#MPC: the number of predicted protein complexes matched by known protein complexes;

#MKC: the number of known complexes matched by predicted protein complexes;

#Perfect: the number of known complexes perfectly matched by predicted protein complexes.

#### Performance of weighted approach

Table 2 also exhibits the validity of our weighted approach. For instance, applying CAMSE algorithm, the *F-measure* obtained from WTEPIN, WDPIN and WSPIN are 36%, 18% and 28% higher than that achieved from TEPIN, DPIN and SPIN, respectively. Applying MCL algorithm, we find a 28% reduction (in contrast to TEPIN) in the number of the predicted protein complexes identified from WTEPIN, which is mainly due to the removal of the edges with negative weight. Nevertheless, the *F-measure* obtained from WTEPIN, WDPIN and WSPIN are 36%, 44% and 39% higher than that obtained from TEPIN, DPIN and SPIN, respectively. In short, our weighted approach dramatically enhances the efficiencies of the PINs, which greatly improves the accuracy of protein complexes identification.

In conclusion, the time-evolving dynamic network TEPIN constructed with our new method can reveal the dynamic evolutionary procedure of protein interactions more precisely than the other networks, which naturally leads the prediction of temporal protein complexes get significantly improved. Moreover, the weighted TEPIN offers powerful support for revealing the biological properties of protein interactions, which further optimizes the detection of protein complexes.

### Analysis of Function Enrichment

We manage to implement the function enrichment analysis to validate the efficiency of our *Deviation Degree* method. Using the tool GO::TermFinder (http://www.yeastgenome.org/cgi-bin/GO/goTermFinder.pl), we calculate the *p-values* of the predicted protein complexes identified from WTEPIN and WDPIN by CAMSE algorithm. The other predicted protein complexes are left out in this section for the reason that they have relatively weaker performance according to previous analyses. Besides, owing to the inconvenience of dealing so many predicted protein complexes, here, only the ones containing at least 20 proteins account for our analysis, which still ensures the fairness of comparisons. As a result, we get 301 and 329 predicted complexes from WTEPIN and WDPIN respectively.

As is shown in Table 3, figures in parentheses are the amounts of the predicted complexes with *p-values* falling into the corresponding intervals, while percentages denote the ratio of those complexes to the total predicted complexes. The proportion of predicted protein complexes with biological significance detected from WTEPIN is up to 98.7%. Despite of an 8.5% reduction in the total number of predicted protein complexes (denoted by #*PC*), the number of the complexes with *p-values* falling into interval [0, E-15) obtained from WTEPIN advances 40% in contrast to WDPIN; while the number and proportion of predicted protein complexes with no or weak biological significance derived from WTEPIN are evidently less than that derived from WDPIN. In short, our WTEPIN has a distinct advantage in statistically significant, indicating our *Deviation Degree* method outperforms three-sigma method in practice for identifying the activities of proteins.

**Table 3:** Function enrichment analysis of predicted protein complexes detected from WTEPIN and WDPIN.

Network	#PC	<E-15	[E-15, E-10)	[E-10, E-5)	[E-5, 0.01)	> = 0.01
**WTEPIN**	301	34.9% (105)	27.6% (83)	27.2% (82)	9.0% (27)	1.3% (4)
**WDPIN**	329	22.8% (75)	25.2% (83)	35.0% (115)	15.2% (50)	1.8% (6)

#PC: the total number of predicted protein complexes.

Table 4 provides ten examples of the predicted protein complexes with very small *p-values* identified from WTEPIN. In each row, the proteins shown in bold are involved in the known protein complex that matches best with the predicted complex, while the additional uninvolved proteins within the predicted protein complex probably share the similar functions with this complex. For instance, for the No.1 predicted protein complex, 6 proteins are not involved in its matched known protein complex, of which 4 proteins (namely *yil021w*, *ygl070c*, *ydr404c* and *yor151c*) share the similar annotations—DNA-directed RNA polymerase—with the real protein complex. The No.6-10 predicted protein complexes are detected from WTEPIN but excluded from WSPIN. We obtain 774 extra predicted protein complexes from our WTEPIN in total, of which 706 (91.2%) with *p-value* less than 0.01, explaining our network is more helpful to analyze the protein interaction networks. Given the incompleteness of known protein complex set, the predicted protein complexes with small *p-value*s are highly likely to be true protein complexes, and our weighted TEPIN provides many novel biological knowledge that cannot be detected from the original SPIN.

**Table 4:** Some examples of the predicted protein complexes with small p-values detected from WTEPIN.

No.	p-value	Predicted protein complex	GO term	*OS*
1	4.37e-44	**YOR224C YPR110C YOR341W YOR116C YNR003C YKL144C YJR063W YJL011C YBR154C YPR190C YPR187W YPR010C YOR207C YNL248C YNL113W** YBR245C YGR005C YIL021W YGL070C YDR404C YOR151C	RNA polymerase activity | AmiGO	0.16
2	1.57e-38	**YCR057C YPR144C YPR137W YPL217C YPL126W YOR310C YOR078W YNR054C YNL132W YNL075W YMR300C YMR128W YMR093W YML130C YLR409C YLR222C YLR197W YLR186W YLR175W YLR129W YLL011W YKR060W YKL099C YJR002W YJL109C YJL069C YJL033W YHR196W YHR169W YHR148W YGR145W YGR128C YGR090W YGL171W YGL120C YER082C YDR449C YDR382W YDR365C YDR324C YDR299W YDL213C YDL148C YDL014W YCL059C YBR247C YBL004W** YNL061W YPL043W YNL207W YLR180W YDL208W YPL012W YHR089C YDR060W YOL010W YPL094C YNL064C YGR210C YDR034C YDR502C	snoRNA binding |AmiGO	0.50
3	1.16e-37	**YPR110C YPR187W YPR010C YOR224C YOR210W YOR207C YOR116C YNR003C YNL248C YNL151C YNL113W YKR025W YKL144C YJR063W YJL011C YDL150W YBR154C** YOR332W YNL308C YNL229C YMR285C YLR086W	DNA-directed RNA polymerase activity |AmiGO	0.45
4	3.15e-37	**YOR116C YPR190C YPR187W YPR110C YOR224C YOR207C YNR003C YNL151C YNL113W YMR116C YKR025W YKL144C YKL060C YJL011C YFR037C YDR045C YDL150W YBR154C YOR341W** YBR245C YMR091C YCR052W YGL070C	RNA polymerase III activity | AmiGO	0.54
5	7.02e-36	**YJR063W YPR187W YPR110C YPR010C YOR341W YOR340C YOR224C YOR210W YNL113W YDR156W YNL248C YBR154C** YBR228W YBR220C YBR187W YOR116C YKL144C YOR207C YNR003C YBR245C	DNA-directed RNA polymerase activity |AmiGO	0.51
6	7.24e-11	**YHR099W YCL010C YBR198C YDR448W YGL066W** YOR244W YNL189W YHR090C YGR002C YFL024C YEL018W YDR392W YDR359C YDR146C	histone acetyltransferase activity | AmiGO	0.16
7	7.17e-08	**YGR252W YOL148C** YDL140C YPR187W YOR224C YOR151C YOL145C YLR418C YJR017C YGR136W YER125W YDR167W YBR279W YMR236W YBR081C	RNA polymerase II activity | AmiGO	0.13
8	3.54e-07	**YIL035C YOR061W YOR039W** YPL235W YPR110C YNL107W YML112W YJL081C YGR274C YGR040W YGL150C YDR243C YDR190C YDL225W YDL002C YBR245C YGL019W	ATP-dependent 3'-5' DNA helicase activity |AmiGO	0.24
9	2.11E-07	**YOL148C YLR055C YDR392W YDR167W YBR081C YMR236W YGR252W** YPR086W YER148W YKR001C YOR151C YDL140C	transcription factor activity, transcription factor binding | AmiGO	0.26
10	2.50E-07	**YDR167W YBR081C YOL148C YDR392W YLR055C** YER148W YPR086W YNL039W YKR001C YFR034C YER164W YBR245C	transcription factor activity, transcription factor binding | AmiGO	0.13

**Fig 5:**
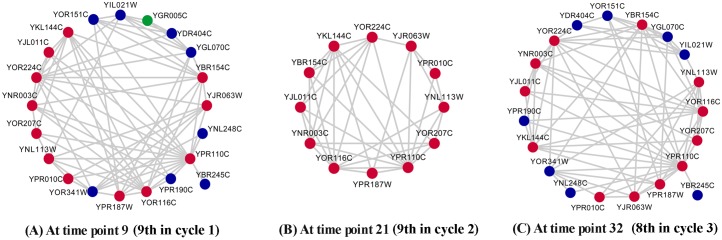
Dynamic evolutionary procedure of a predicted temporal protein complex. The red proteins are unchanged in this procedure; the blue ones shown in (A) are absent in (B), and then reappear in (C); and the green protein shown in (A) is absent in both (B) and (C).

Fig 5 illustrates the dynamic evolutionary procedure of the first predicted temporal protein complex shown in Table 4. This protein complex exactly share five Gene Ontology terms—such as RNA polymerase activity | AmiGO with the lowest *p-values—*under three different time points, meaning this predicted complex can perform five different biological functions. We analyze the active time points of 21 proteins involved in this predicted protein complex. After the disassembly of the complex at time point 9 (Fig 5(A)), eight proteins are reactivated at other time points to perform functions with their partners (not shown), namely *ygl070c*, *yil021w*, *ygr005c*, *ybr245c*, *yor151c*, *ydr404c*, *yor341w* and *ypr190c*; while other 12 proteins are reassembled at time point 21 to form the original protein complex, namely *yor224c*, *ypr187w*, *yor116c*, *ynr003c*, *ykl144c*, *yjr063w*, *yjl011c*, *ybr154c*, *ypr010c*, *yor207c*, *ynl113w* and *ypr110c*, which is shown in Fig 5(B). At time point 32, except *ygr005c*, all of these 21 proteins are assembled again to form a protein complex with the same Gene Ontology terms as before, which is shown in Fig 5(C). Such a progress reveals the dynamic assembly process of protein complex. In addition, as we know that each cycle of yeasts’ gene expression data GSE3431 contains 12 time points, from this example we can see that the protein complex is always assembled at the 8^th^ or 9^th^ time point in each metabolic cycle, thus the changing process of this protein complex reflects the periodicity of yeasts’ metabolism.

## Conclusions

Protein complex is a fundamental unit formed with highly connected proteins and often possesses specific biological functions [26]. In biology, protein interaction networks (PINs) are not static—they dynamically change over time and are responsive to the stimuli caused by external environment. Nevertheless, the static PINs couldn’t inform us temporal and contextual signals. As temporal protein complexes can better reflect the real-world dynamic molecular mechanisms inside the cellular systems [27], it is crucial to construct time-evolving dynamic PINs to reveal the dynamics within PINs. Although a few available dynamic PINs perform well in practice for mining temporal protein complexes, they often involuntarily exclude many proteins with low or high expression levels, which lead the dynamics in PINs cannot be revealed effectively.

In this paper, we develop a *Deviation Degree* method with capability to successfully identify the active time points of proteins based on the deviation degree of gene expression curves. We construct a time-evolving PIN (TEPIN) which eliminates the disadvantages in other methods for constructing dynamic PINs. Further, we weight the TEPIN to depict the biological properties of protein interactions, as well as to diminish the impacts of the inherent false negatives and false positives in PINs. The experimental results show that the predictions of protein complexes on TEPIN outperform those on the other networks in terms of various evaluation measurements, which indicates the approach can reveal the dynamic evolutionary procedure of protein interactions more correctly than the other networks. Moreover, the weighted TEPIN further optimizes the detection of protein complexes. We obtain huge amount of predicted protein complexes with strong biological significance and provide helpful biological knowledge to the relate researchers. In addition, our analysis of the dynamic evolutionary procedure of a predicted temporal protein complex verifies the fact that protein complexes are assembled just-in-time.

Time-evolving dynamic PIN eliminates the noises which exist in static PIN and provides increased reliability for uncovering the dynamic protein assembly progress for cellular organization [28]. Therefore, it has important implications to our knowledge of the dynamic organization characteristics in cellular systems to construct effective dynamic PIN.
